# An Extended Network of Genomic Maintenance in the Archaeon *Pyrococcus abyssi* Highlights Unexpected Associations between Eucaryotic Homologs

**DOI:** 10.1371/journal.pone.0079707

**Published:** 2013-11-07

**Authors:** Pierre-François Pluchon, Thomas Fouqueau, Christophe Crezé, Sébastien Laurent, Julien Briffotaux, Gaëlle Hogrel, Adeline Palud, Ghislaine Henneke, Anne Godfroy, Winfried Hausner, Michael Thomm, Jacques Nicolas, Didier Flament

**Affiliations:** 1 Ifremer, UMR6197, Laboratoire de Microbiologie des Environnements Extrêmes, Plouzané, France; 2 Université de Bretagne Occidentale, UMR6197, Laboratoire de Microbiologie des Environnements Extrêmes, Plouzané, France; 3 CNRS, UMR6197, Laboratoire de Microbiologie des Environnements Extrêmes, Plouzané, France; 4 Lehrstuhl für Mikrobiologie, Universität Regensburg, Regensburg, Germany; 5 IRISA-INRIA, Campus de Beaulieu, Rennes, France; Max-Planck-Institute for Terrestrial Microbiology, Germany

## Abstract

In Archaea, the proteins involved in the genetic information processing pathways, including DNA replication, transcription, and translation, share strong similarities with those of eukaryotes. Characterizations of components of the eukaryotic-type replication machinery complex provided many interesting insights into DNA replication in both domains. In contrast, DNA repair processes of hyperthermophilic archaea are less well understood and very little is known about the intertwining between DNA synthesis, repair and recombination pathways. The development of genetic system in hyperthermophilic archaea is still at a modest stage hampering the use of complementary approaches of reverse genetics and biochemistry to elucidate the function of new candidate DNA repair gene. To gain insights into genomic maintenance processes in hyperthermophilic archaea, a protein-interaction network centred on informational processes of *Pyrococcus abyssi* was generated by affinity purification coupled with mass spectrometry. The network consists of 132 interactions linking 87 proteins. These interactions give insights into the connections of DNA replication with recombination and repair, leading to the discovery of new archaeal components and of associations between eucaryotic homologs. Although this approach did not allow us to clearly delineate new DNA pathways, it provided numerous clues towards the function of new molecular complexes with the potential to better understand genomic maintenance processes in hyperthermophilic archaea. Among others, we found new potential partners of the replication clamp and demonstrated that the single strand DNA binding protein, Replication Protein A, enhances the transcription rate, *in vitro*, of RNA polymerase. This interaction map provides a valuable tool to explore new aspects of genome integrity in Archaea and also potentially in Eucaryotes.

## Introduction

The control of genome integrity is of pivotal importance for the cell. Failure to maintain genome stability results in the accumulation of mutations, genome rearrangements and cell death. Molecular interactions involved in DNA repair and recombination and their coordination with DNA synthesis are required for genome maintenance. These genome transactions and their proper coordination require the assembly of multi-protein machines at specific sites at the chromosome or chromatin level. The wide variety of lesions that can arise in DNA and the biological importance of coping with these are reflected by the diversity of biochemical strategies for DNA repair found in model micro-organisms. In this context, Hyperthermophilic Archaea (HA) are of special interest as DNA in hyperthermophiles is exposed to temperatures that increase the rate of decomposition [1]. However, in the hyperthermophilic euryarchaeon *P. abyssi*, the level of apurinic/apyrimidinic sites was shown to be only 10-fold higher than in *E. coli* [2] and the level of spontaneous mutation in HA has been shown to be close to the standard rate for model microbes [3], suggesting active and efficient repair pathways. In addition to this tolerance to extreme temperature, HA have a deep evolutionary divergence from well-studied micro-organisms. How they maintain genomic integrity is thus an interesting question from both physiological and phylogenetic perspectives [4]. However, very little is known concerning pathways and proteins that perform DNA repair in HA. Genomic sequence analyses suggest that most of the archaeal proteins predicted to participate in DNA replication, repair and recombination are more closely related to eucaryotic than bacterial proteins [5,6].

So far, proteins of base excision repair, alkyl transfer, damage reversion and trans-lesion pathways have been detected in HA [7,8,9,10]. In contrast, genomic content analysis show that mismatch repair (MMR) machinery is absent in most archaea species. The presence of the *mutS/mutL* ortholog genes of the MMR pathway is restricted exclusively to halophiles and methanogen species and has been shown to be dispensable for the maintenance of a low mutation rate in *Halobacterium salinarum* NRC-1 [11]. In this context, the low mutation rate observed could imply that Archaea display mechanisms that have evolved to improve nucleotide insertion and proofreading activities during DNA replication or that a distinct MMR, which has eluded genomic analysis, operates in Archaea. Furthermore, the existence of nucleotide excision repair (NER) pathway has not been demonstrated, yet it is suggested by the presence and properties of NER nucleases and helicases in HA. However, none of the proteins responsible for the lesion recognition step have been found in archaeal genomes [6]. In this context, the implication of new components or new DNA repair pathways cannot be ruled out. Another conspicuous feature highlighted by genomic analysis is the relative paucity of DNA polymerases dedicated to repair functions in archaeal genomes which seems inconsistent with the lifestyle of these micro-organisms at high temperatures and raises the hypothesis that archaeal replicative DNA polymerases could boast additional repair functions alone or associated with other proteins. Many aspects of DNA repair and more generally regulation of genomic maintenance thus remain enigmatic. To gain insight into these fundamental processes, genetic systems in Archaea have been developed and a wide range of genes involved in DNA replication and repair have been disrupted in order to understand their physiological functions in HA [12]. However, to date, very few reports applied this functional approach to the analysis of DNA repair in anaerobic HA [13,14]. In addition, due to the difficulty to cultivate these extremophiles organisms, the genetic manipulation of HA species is still in its infancy. Collectively, these statements call for the development of additional approaches to explore these mechanisms with the potential to provide new functional hypothesis to test using the complementary approaches of genetics and biochemistry

As an alternative, identification of protein-protein interaction networks in organisms can complement information obtained from these functional genomic approaches. Indeed, to fulfill their biological activities in the cell, most proteins function in association with protein partners, or as part of large molecular assemblies. Therefore, the knowledge of the interaction context of a protein is crucial to understand its cellular functions. Two previous studies reported the elucidation of protein-protein networks of archaeal DNA replication, using yeast two-hybrid screens or purification of stable complexes assembled *in vivo*, in *Archaeoglobus fulgidus* and *Thermococcus kodakarensis*, respectively [14,15]. The latter study led to the important discovery and characterization of a novel GINS-associated nuclease, GAN, which might be the archaeal homologue of Cdc45 in the CMG complex [16,17]. 

Can we complete or better define the repertoire of proteins involved in genomic integrity pathways and what are the connections between informational processes in Archaea ? To answer these questions, we delineated a protein-protein interaction network of genomic maintenance in *P. abyssi* using affinity purification *in vitro* coupled with mass spectrometry identification of protein partners (AP/MS). Compared to the purification of protein complexes *in vivo*, this approach allows the purification of proteins independently of the cellular concentrations and compartmentalization of the bait proteins. However, the stoichiometry of interacting proteins is not maintained and the high concentration of bait proteins used can lead to false positive identifications. To reduce the effect of false positive identifications, AP/MS assays were carried out in duplicate, for each bait, and the results compared against five independent negative controls. In addition, we used a simple algorithm to provide clear cut results of the mass spectrometry identification. 23 HIS or GST-tagged proteins presumably implicated in DNA replication, repair and recombination were used to co-isolate protein complexes [18], allowing us to propose an extended network of genome stability at high temperature in *P. abyssi*. For some of the interactions detected, we demonstrated physical associations, using surface plasmon resonance and pulldown-immunoprecipitation assays or a functional association using an enzymatic assay, as exemplified by the activation of transcription in the presence of a RPA complex. Interestingly, using this approach we revealed and confirmed unsuspected interactions between eucaryotic homologs.

## Materials and Methods

### *Pyrococcus abyssi* protein extract

*P. abyssi* was grown in continuous culture in a 5 L Gazlift bioreactor in MES medium under anaerobic conditions at 95°C and pH 6.4 [19]. Cells were maintained in exponential growth phase between 2 and 4 x 10^8^ cells.ml^-1^. Cell culture was harvested on ice. Cells were pelleted at 6000 g, 45 min at 4°C and dry cell pellets were stored at -80°C. *P. abyssi* cells pellets were resuspended in two volume (w/v) of PBS buffer supplemented with 10 mM imidazole and a mix of EDTA-free protease inhibitor (Roche). Cell lysis was completed by sonication using a Vibracell sonifier (BioBlock Scientific, 2 x 10 min of 0.5 second on/off pulses at 375 W, 40% amplitude, on ice). *P. abyssi* cells debris were eliminated by centrifugation at 15000 g, 45 min at 4°C. Soluble fraction concentrations were measured by Dc Protein Assay (Bio-Rad) with BSA as the standard and adjusted at 8 mg.ml^-1^ and pH 7.4.

### Production and purification

The production and purification of the bait proteins were either already reported (see Table 1 for a listing of the protein baits used and the associated references) or are described in Materials S1. The sequences of the inserts were checked and the recombinant clones were used to transform *E. coli* Rosetta^TM^ strain. *E. coli* clones were grown in 300 mL LB medium. Protein production was induced in exponential growth phase at OD_600nm_ = 0.6 by the addition of 1 mM IPTG, 3h at 37°C. Cell lysis was performed by applying 3 successive heat shock (10 min at 80°C) for HIS tagged proteins and by sonication for GST tagged proteins, using a Vibracell sonifier (10 min of 0.5 second on/off pulses at 375 W, 40% amplitude). Cell lysates were cleared by centrifugation at 10000 g, 10 min at 4°C. Recombinant proteins were purified from soluble fraction on magnetic beads (Dynabeads® His-Tag Isolation & Pulldown or Promega MagneGST™ Pull-Down System). Two milligrams of beads, equilibrated in PBS buffer were incubated 15 min at room temperature under rotation with 4 ml of cell lysate. Purified proteins were washed 4 times with 1mL of PBS buffer. Quality of the purification and apparent size of the protein were checked on SDS-PAGE. 

**Table 1:** List of bait proteins used in affinity purification experiments.

**Protein**	**Biological process**	**Tag/location**	**Production/Purification^a^**
MCM (*Q9UYR7*)	Replicative helicase	HIS/N-ter	FL, Materials S1
Gins 23 (*Q9UYR8*)	Replication	HIS/N-ter	FL [18]
Gins 51 (*Q9UYX9*)	Replication	HIS/N-ter	FL [18]
Rec-J like exonuclease (*Q9V2K8*)	Gins Associated Nuclease	HIS/N-ter	FL [18]
PCNA (*Q9UYX8*)	Replication/Repair	HIS/N-ter	FL [24]
DNA Polymerase B (*P0CL76*)	Replication	HIS/N-ter	FL [72]
DNA Polymerase D (*Q9V2F3, Q9V2F4*)	Replication	HIS/N-ter (ssu)	FL complex [2]
Primase small subunit P41 (*Q9V292*)	Replication	HIS/N-ter	FL [73]
Primase large subunit P46 (*Q9V291*)	Replication	HIS/N-ter	FL [73]
DNA ligase (*P0CL74*)	Replication /Repair	HIS/N-ter	FL [24]
Fen1 (*Q9V0P9*)	Replication/Repair	HIS/N-ter	FL [24]
Ribonuclease HII (*Q9V1A9*)	Replication/Repair	HIS/N-ter	FL [74]
DNA2 homolog (*Q9V2G5*)	Helicase/nuclease of unknown function	HIS/N-ter	C-ter domain 1070K-1308 [18]
Replication Protein A (*Q9V1Z1, Q9V1Y9, Q9V1Z0*)	Replication/Repair/Recombination	HIS/N-ter (RPA41)	FL complex, Materials S1
NucS (*Q9V2E8*)	Repair	HIS/N-ter	FL [75]
Mre11/Rad50 complex (*Q9UZC9, Q9UZC8*)	Recombination/Repair	HIS/N-ter (Mre11)	FL complex, Materials S1
RadA (*Q9V233*)	Recombination	HIS/N-ter	FL [18]
RadB (*Q9V2F6*)	Recombination	HIS/N-ter	FL [18]
alkA DNA glycosylase (*Q9UZ73*)	Repair	HIS/N-ter	FL [18]
OGG1 N-glycosylase/DNA lyase (*Q9UZY0*)	Repair	GST/N-ter	FL [18]
NAD-dependant protein deacetylase (*Q9UZE7*)	Chromatin protein acetylation	HIS/N-ter	FL [18]
php domain containing protein (*Q9V0F9*)	Unknown	HIS/N-ter	FL [18]
Rad-25 like (*Q9V278*)	helicase of unknown function	HIS/N-ter	FL [18]

a FL: full length.

### Affinity purification of *P. abyssi* protein complexes

Purified HIS and GST tagged proteins were used as baits in the *P. abyssi* protein extract. Depending on the tagged proteins sizes and binding properties, between 10 and 30 µg of bait proteins were immobilized on 2 mg of either cobalt or gluthatione coated magnetic beads. The complex baits-beads were further incubated with 4 ml of *P. abyssi* protein extract under rotation for 3 hours at 4°C. Protein complexes formed *in vitro* were separated on a magnet and washed extensively by 4 x 4 ml and 2 x 1 ml of PBS buffer. 

A second assay was performed with an additional step of nucleotide degradation to eliminate non-specific protein interactions *via* DNA or RNA fragments. To this aim, purified complexes were incubated with 80/1.2 units of a DNAse/RNAse mix (GE healthcare) for 45 minutes in PBS buffer at room temperature. Those two affinity purification experiments will be referred to as nuc- and nuc+, respectively. Finally, a control assay was also performed under identical conditions using glutathione- and cobalt-coated magnetic beads in place of the baits-beads complexes. Five such independent control assays were done to identify the proteins engaged in non-specific interactions with the beads. Purified protein complexes were eluted in 100 µL of Laemmli buffer at 95°C for 15 min and separated *via* SDS-PAGE for 20 min at 120V on 12% Criterion XT Precast Gels (Bio-Rad). Protein extraction was carried out by dividing each protein track into three equally sized segments, followed by shotgun proteomic analysis of each gel segment. 

### Protein prefractionation and digestion

The mass spectrometry analyses were performed at the BioGenOuest proteomics core facility (http://www.biogenouest.org/en/content/platform/proteomics). The gel slices were first treated with 50 mM NH_4_HCO_3_ in acetonitrile/water 1:1 (v/v), dehydrated with 100 % acetonitrile and rehydrated in 100 mM NH_4_HCO_3_, washed again with 50 mM NH_4_HCO_3_ in acetonitrile/water, 1:1 (v/v) and finally dehydrated with 100 % acetonitrile. The slices were then treated with 65 mM DTT for 15 minutes at 37°C, and with 135 mM iodoacetamide in the dark at room temperature. The samples were washed with 100 mM NH_4_HCO_3_ in acetonitrile/water, 1:1 (v/v), and dehydrated with 100 % acetonitrile before being rehydrated in 100 mM NH_4_HCO_3_, washed with 100 mM NH_4_HCO_3_ in acetonitrile/water, 1:1 (v/v) and then dehydrated again with 100% acetonitrile. Proteins were digested overnight at 37°C with 4 ng/µl of modified trypsin (Promega) in 50 mM NH_4_HCO_3_. Peptides were extracted by first incubating the slices in 80 µl of acetonitrile/water/formic acid (70/30/0.1; v/v/v) for 20 minutes, and then in 40 µl of 100% acetonitrile for 5 minutes and finally in 40 µl of acetonitrile/water/formic acid (70/30/0.1; v/v/v) for 15 minutes. Supernatants were transferred into fresh tubes and concentrated in a SpeedVac (Thermo Scientific) for 15 minutes to a final volume of 40 µl.

### Mass spectrometry analysis

The MS measurements were done with a nanoflow high-performance liquid chromatography (HPLC) system (Dionex, LC Packings Ultimate 3000) connected to a hybrid LTQ-OrbiTrap XL (Thermo Fisher Scientific) equipped with a nanoelectrospray ion source (New Objective). The buffers used for chromatography were 0.1% formic acid (buffer A) and 100% acetonitrile/0.1% formic acid (buffer B). To prevent cross-contaminations between different affinity purification sets, the control assay was first applied on the HPLC column followed by the nuc+ and the nuc- assay of each bait. 10 µl of prepared peptide mixture was loaded on a trapping precolumn (5 mm × 300 μm i.d., 300 Å pore size, Pepmap C18, 5 μm) for 3 minutes in 2% buffer B at a flow rate of 25 µl/minute. This step was followed by reverse-phase separation at a flow rate of 0.250 µl/minute using an analytical column (15 cm × 300 μm i.d., 300 Å pore size, Pepmap C18, 5 μm, Dionex, LC Packings) with a gradient ranging from 2% to 35% buffer B for the first 60 minutes, 35% to 60% buffer B from 60 to 85 minutes, and 60% to 90% buffer B from 85 to 105 minutes. The peptides were detected by directly eluting them from the HPLC column into the electrospray ion source of the mass spectrometer. An ESI voltage of 1.5 kV was applied to the HPLC buffer using the liquid junction provided by the nanoelectrospray ion. Survey full scan MS spectra (mass range 400 - 2000) were acquired in the OrbiTrap section of the instrument with a resolution of R = 60’000 at m/z 400; ion injection times were calculated for each spectrum to allow for accumulation of 106 ions in the OrbiTrap. The seven most intense peptide ions in each survey scan with an intensity above 2000 counts and a charge state ≥2 were sequentially isolated at a target value of 10’000 and fragmented in the linear ion trap by collision induced dissociation (CID). Normalized collision energy was set to 35 % with an activation time of 30 milliseconds. Peaks selected for fragmentation were automatically put on a dynamic exclusion list for 120 seconds with a mass tolerance of +/- 10 ppm to avoid selecting the same ion for fragmentation more than once. For an optimal duty cycle the fragment ion spectra were recorded in the LTQ mass spectrometer in parallel with the OrbiTrap full scan detection. For OrbiTrap measurements, an external calibration was used before each injection series ensuring an overall error mass accuracy below 5 ppm for the detected peptides. MS data were saved in RAW file format (Thermo Fisher Scientific) using XCalibur 2.0.7 with tune 2.4.

### Data processing and identification of peptides and proteins

The data analysis was performed with the Proteome Discoverer 1.2 software supported by Mascot (Matrixscience) and SEQUEST database search engines for peptide and protein identification. MS/MS spectra were search against The SwissProt Database filtered with the *Pyrococcus abyssi* taxonomy (release-2010_10, 1812 sequences, 545621 residues), for protein identification using the Proteome Discoverer software (version 1.2.0.208). Mass tolerance for MS and MS/MS was set at 10 ppm and 0.5 Dalton, respectively. The enzyme selectivity was set to full trypsin with one miss cleavage allowed. Identified peptides were filtered based on Xcorr values and the Mascot score to obtain a false discovery rate of 1 %. 

### Identification of specific interactions

The MS data were processed manually in order to identify bait-prey specific interaction signals. To rationalize this process, we considered that the identification of a protein was significant when, at least, 5 independent peptides covering at least 10% of the protein sequence were identified and that the resulting mascot score (M) was over 100. We then defined a noise/signal significance ratio (SR) for each identified proteins. SR was calculated by dividing the independent control assay maximal Mascot scores (M_neg_) by the pull-down Mascot scores M_nuc-_ or M_nuc+_. Proteins that never appeared in the control assays were arbitrarily given a M_neg_ value of 20. We set the significance threshold at a SR score of 0.2 (M_nuc+/-_ ≥ 5 X M_neg_) ; an interaction was considered to be of reasonable confidence when both SR_nuc-_ and SR_nuc+_ were below 0.2. The resulting interaction network was visualized using cytoscape software [20].

### Protein Network and Bioinformatics analyses

#### The network

The protein-protein interaction network is enriched by associating to each node GO terms and Interpro domains extracted from available protein annotations in UniProt database (http://www.uniprot.org/). Furthermore, we used the Domine database v2.0 [21] for the prediction of domain interactions with an associated confidence level. A synonym table was used to translate Interpro domains into Pfam domains and confidence values of interactions were translated into discrete levels according to the following coding scheme: NA=1, LC=2, MC=4, HC=5.

#### Topology of the network

The *clustering coefficient* of a node (protein) is computed as the proportion of links observed between its direct neighbors (proteins interacting with it) with respect to the maximum possible number of links they could share. It takes value 1 for a clique (complete set of interactions) and 0 for a star (protein connected to a set of independent proteins). In case of bipartite graphs like protein-protein interaction networks (only a subset of real interactions can be observed, those between a bait and a prey), the ratio is made with respect to the cross product of baits and preys. Main *clusters* of the network have been extracted according to the following procedure filtering pseudo-cliques in the network: all proteins whose clustering coefficient exceeds a stringent threshold (20% in the network) are first extracted and form the basis of a cluster with the subset of their interacting partners. In a second step, the proteins that are supported by only one interaction in a cluster are discarded, together with all clusters that are included in a larger cluster.

Another important aspect of the network structure analysis is to try to distinguish direct interactions from indirect ones. The aim is to obtain a scaffold, a spine or a backbone of the network that provides an abstract view of the distances between proteins in terms of communication between subparts of the network. Minimal Spanning Trees (MST) have been recognized as a good means to unravel the intricate structure emerging from affinity purification data [22]. A spanning tree is a tree connecting all nodes in a graph. If the edges are weighted, it is possible to compute a MST, that is, a spanning tree with a minimal total weight. In an interaction network however, not all proteins are equal and considering the whole set of proteins may hinder the search for its core spine. The proteins having a topologically central place have been shown to play a more important biological role than others on the human PPI network [23], using the notion of *Minimum Connected Dominating Set* (MCDS). A connected dominating set in a graph is a subset of its nodes such that they are connected and there exists an edge between each remaining node and a node in this subset. A MCDS has furthermore a minimal size. We have combined the two concepts by looking for a minimal spanning tree on the subgraphs of minimum connected dominating sets (i.e, a MST with respect to all possible MCDS). Thus, the produced backbone is a minimal weighted tree of interacting proteins such that all other proteins have a direct interaction with one of its proteins. In order to get the best support in the data, we use both the interaction network and the network of known associated interacting domains (domine). Three criteria are cumulated in the weight: the SR (highest priority), the opposite of the associated domain confidence, and the domain number (lowest priority).

### Pull-down immunoprecipitation assays

For the interaction PCNA:Pab0431, Pull-down experiments were performed as described earlier [24,25] with the exception that 5µg of Pab0431 was incubated with 1 mg of Co^2+^ coated magnetic microbeads (Invitrogen) and that the beads-baits complexes formed were then incubated with 16.5 mg of *P. abyssi* cell extract for 2 h at 4 °C. 

For the interaction PCNA:Mre11/Rad50, 10 µg of Mre11:Rad50 complex was immobilized on 240 µg of magnetic microbeads (Invitrogen) in 200 µL of binding buffer (TrisHCl 20 mM pH 8, NaCl 150 mM, Dithiothreitol 1 mM) in the presence of 20 µg of BSA. When indicated, DNA was degraded by the addition of 5 U of DnaseI (Sigma) in 200 µL of TrisHCl 50 mM pH 8, MgCl_2_ 4 mM for 1 h at room temperature. After 3 washing steps in binding buffer, 0.5 µg of PCNA was added for 1 h at room temperature in 200 µl of binding buffer in the presence of 30 µg of BSA. After 3 final washing steps, the proteins were eluted from the beads in Laemmli buffer 5 minutes at 95°C and separated on SDS PAGE (4-20%, Pierce). Proteins were transferred onto a PVDF membrane (Thermo Scientific). PCNA and Mre11 were probed using a polyclonal anti-PCNA and monoclonal anti-HisTag antibodies (Invitrogen), respectively. Dylight fluorophore coupled secondary antibodies (Thermo Scientific) were used to reveal the proteins. Fluorescence was detected using a Typhoon (Amersham Bioscience) imager and analysed with ImageQuant software.

### *In vitro* transcription assay

*In vitro* transcription reactions were performed as described previously [26]. For basal transcription, reaction mixtures were assembled in 25 µl (final volume) of transcription buffer (40 mM HEPES-Na pH 7.3, 250 mM NaCl, 2.5 mM MgCl2, 0.1 mM ZnSO4, 0.1 mM EDTA, 0.1 mg/ml BSA, 5% Glycerol). 10 nM gdh-C20 template DNA was combined with 100 nM TBP, 35 nM TFB, and 10 nM RNAP. Transcription was initiated by the addition of ATP, CTP, and GTP to a final concentration of 40 µM and UTP to a final concentration of 2.7 µM, including 2 µCi of [α-32P]UTP (3000 Ci/mmol). After incubation at 70 °C for 15 min, the transcripts were analyzed by denaturing PAGE. The reaction products were quantified using AIDA image analysis software, using only the areas corresponding to the define bands of the run-off transcripts. The activation fold was determined by comparison with the value of the control without RPA or RPA buffer (lane 1).

### Surface plasmon resonance

Data were acquired at 25°C using Reichert SR7000DC spectrometer instrument (Reichert Inc., Buffalo, USA). Running buffer was PBS Tween 0.5% and the flow rate was 20 µL.min^-1^. Streptavidin (Sigma, France) was immobilized on a mixed SAM (1 C_11_-(OEG)_6_-COOH :10 C_11_-(OEG)_3_-OH) surface *via* classical amine coupling chemistry. Then, 66 µRIU of the 5’ biotinylated *ss*DNA 5’-TGCCAAGCTTGCATGCCTGCAGGTCGACTCTA-3’ was immobilized on the biotin surface. Each measurement cycle consisted first in a saturation of the *ss*DNA chip by RPA (10 min at 100 nM) followed by an injection of the different analytes at the following concentrations: 16.67 nM, 50 nM, 150 nM and 450 nM. Regeneration of the sensor was achieved with 3 injections of 0.5 % SDS for 60 sec. Each curve was referenced against *ss*DNA/RPA dissociation curves. Data were then fitted using a global analysis method with Scrubber 2.0a software (Biologic Software, Australia).

## Results and Discussion

Twenty three recombinant proteins (Table 1), protein complexes and truncated domains of proteins, were produced, purified and used as molecular baits to look for potential interacting partners in the cell extracts of *P. abyssi*, using AP/MS identification analysis (Figure S1). These proteins were either documented or predicted to be implicated in genome maintenance processes in Archaea (see Table 1 for references). To avoid non-specific interactions mediated by nucleotidic substrates, we performed two distinct pull-down experiments per bait, in the presence or absence of RNA/DNA nucleases. The proteins identified by mass spectrometry, that were present in both AP/MS experiments and for which the Mascot score was more than 5 fold higher in the assays compared to the negative controls, were regarded as being able to specifically interact with the bait under consideration. This corresponded to SR values below the threshold of 0.2 (see materials and methods section). We reasoned that this exploratory analysis should unravel new and unsuspected relationships between known factors and novel components involved in nucleic acid processes, as well as potential moonlighting proteins linking DNA maintenance with distinct metabolic pathways and thus we only used the objective criterion of SR score to classify the proteins identified in mass spectrometry, rather than excluding proteins based on functional annotations. As such, some non-specific interactions might remain in the network depicted, but we believe that this non informative part had been reduced to the minimum thanks to the analysis of two independent AP/MS experiments per bait. In support of this, the results include 72% of proteins described or predicted to act in nucleic acid pathways and contain already documented interactions between established protein partners. In addition, we also identified interactions that have already been described in Eucaryotes but as yet undiscovered in Archaea (see following sections). The putative interactions detected with the highest degree of confidence with and without nuclease treatment, are presented in Table S1. 

### Global Network Description

**Figure 1:**
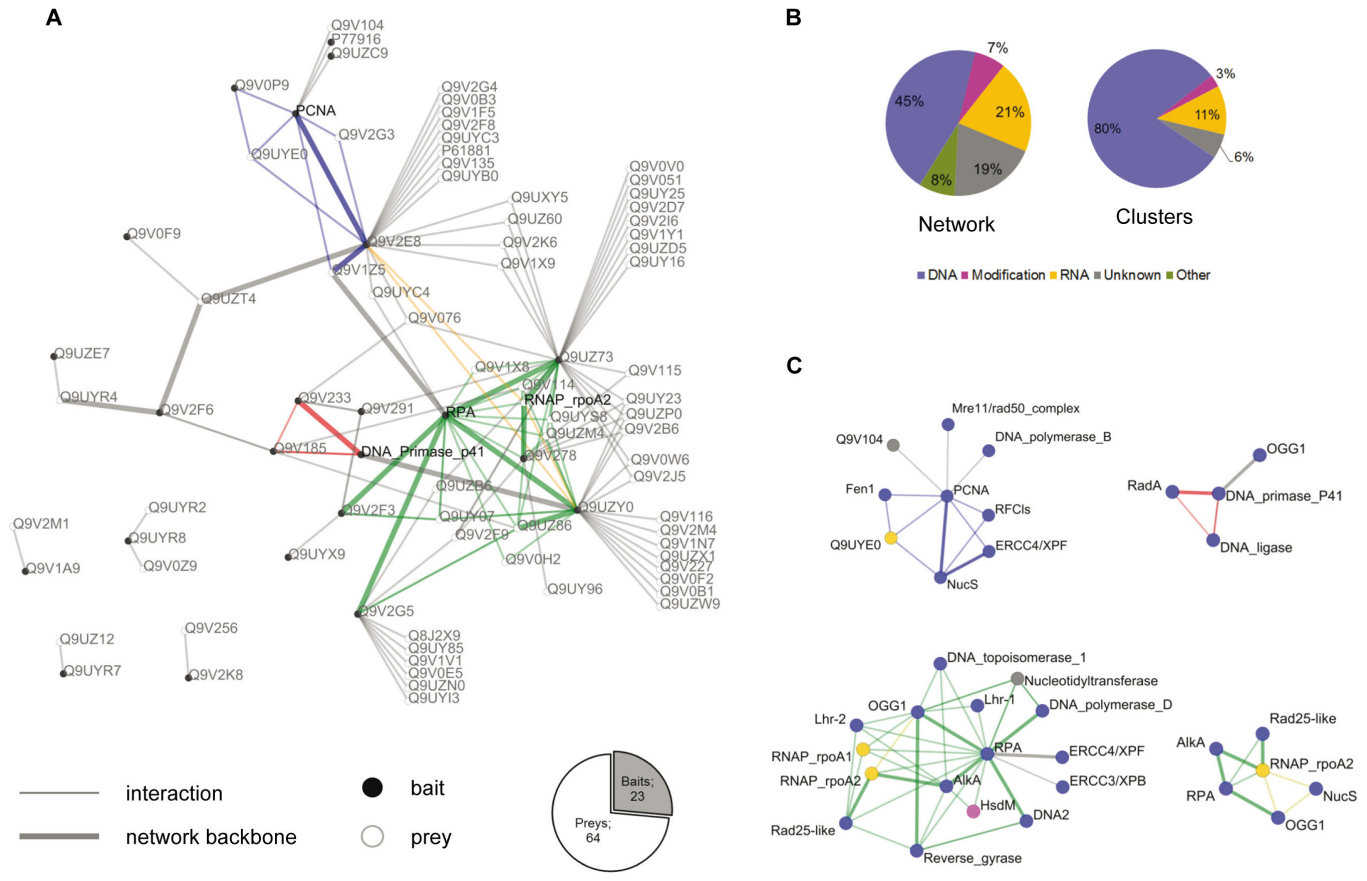
Statistical and topological analysis of the protein interaction network. **A**, protein interaction map. Baits are indicated in black dots and preys as open circles. Network backbone is represented in thick lines. Protein clusters are highlighted in blue (PCNA), Green (RPA) Yellow (RNAP rpoa2) and red (DNA primase p41). **B**, clusters. Nodes colour follow attributed function of represented protein; DNA replication/recombination/repair in blue, RNA metabolisms in yellow, Methylation/modification in pink and unknown functions in grey. **C**, metabolic repartition in the full network and in clusters.

In order to understand the data more thoroughly, the high confidence data set was computed in a network. Graph analysis helped us to identify the main features of the network such as clusters, protein groups and network backbone (Figure 1A). Proteins were represented as nodes and putative interactions as edges, following a spoke model [27]. The network was composed of 87 nodes and 132 edges. Baits represented 26% of the total number of nodes. 66% of the nodes had an established or predicted role in nucleic acid metabolisms and 19% corresponded to uncharacterized functions (Figure 1C). Modification enzymes and other metabolisms represented respectively 7% and 8% of the proteins. In addition, 45% of the interactions linked proteins of the DNA replication/repair/recombination functional categories, which also indicated that the fraction of false positives has been strongly reduced after filtering of the AP/MS data [28]. The network presented an average clustering coefficient of 10 % corrected to 11 % when the bias of prey-prey interactions blindness was taken into account. Dna2-like helicase (Q9V2G5) had the lowest clustering coefficient (0.8%) which suggested, that this protein likely interacts with different partners to form distinct complexes, rather than a unique macromolecular assembly. In contrast, the proteins that are linked to a highly connected cluster and thus most likely interact inside a protein complex (clust. coef. ≥50%, cluster size ≥4), are the DNA topoisomerase 1 and the Lhr-2 protein, which are both related to the Alka-OGG1-RPA set of proteins. Using graph analysis, we also identified highly connected areas in the network i.e. the optimal cliques of at least 3 proteins in the network (Figure 1B). 

The clustering centers were Proliferating Cell Nuclear Antigen homotrimer (PCNA), Replication Protein A heterotrimer (RPA), the catalytic subunit of DNA primase p41 and RNA polymerase subunit A2. In addition, we also computed the minimum spanning tree (MST) of the dominating set of the network, using first the SR scores and then the presence of known interacting domains as the constraints. This sub-graph (edges highlighted in thick lines cf Figure 1) corresponded to a backbone network that contained only the essential topological information to represent the protein network, based on the consistency of the AP/MS analysis and on the presence of interacting domains, as described in domine database (see materials and methods section). It represented the interactions that are more likely direct within the network. This sub-tree presented a marked root corresponding to the RPA complex. Altogether, the clustering and MST analysis emphasized the fact that PCNA and RPA complexes are essential proteins in Archaea, involved in distinct and numerous DNA transactions, spanning DNA replication, DNA repair and DNA recombination, and that both factors are involved in interactions with many different partners [29,30,31,32,33].

### Are the interactions detected specific and biologically relevant?

Others have already attempted to reveal the interaction network involved in Archaeal DNA replication using either Y2H and AP/MS *in vivo* approaches, in *Archaeoglobus fulgidus* and *Thermococcus kodakaraensis*, respectivelty [14,15]. We compared our data to these studies and found few common interactions (Table 2), representing 6.6 and 8 % of overlap when we only considered the interactions detected by the same baits, used by Motz et al and Li et al, respectively. The previous studies used different methodological approaches on different species to determine potential physical associations, which probably accounts for the differences in coverage observed [28]. However, we took advantage of these two datasets to analyze our own raw data in greater depth. We were thus able to identify two additional potential associations, also found by Moltz and co-workers that were originally considered of moderate confidence in our dataset, based on the SR score only; these results will be discussed later (see following sections). Only two associations were common to the three studies, PCNA-Fen1 and PCNA-RFC, likely reflecting the high affinities between these components and their high abundance in the cells.

**Table 2:** Common interactions in protein-protein interaction networks of DNA pathways in Archaea.

Interaction	Datasets *
PolD ssu-PolD lsu	1,2,3
PCNA - Fen-1	1,2,3
PCNA - RFC	1,2,3
PCNA - PolD	1,2
RPA - Q9V1R8	1,2
RPA - Nth endonuclease III	1,2
RPA - Reverse gyrase	2,3
GINS51 - PolD	2,3

* 1, Motz et al. 2002 (Y2H) ; 2, Li et al. 2010 (*in vivo* AP/MS) ; 3, this study (*in vitro* AP/MS).

Comparisons of our data with previously established or reported interactions between components of genomic maintenance in Archaea confirm a large number of the positive results seen in our data. For example, we have shown that the PCNA replication clamp interacted with the small subunit of the loading factor RFC, the replicative DNA polymerase B and the flap endonuclease Fen1. These corresponding interactions have been shown to take place within the chromosome replication machinery [24,34]. In addition, we also found the PCNA associated with the recently described ssDNA nuclease NucS, whilst NucS was able to interact with Hef/XPF protein (Figure 1B). We have previously reported these associations using different technical approaches, and proposed that they could be implicated in branched-DNA repair pathway [25,35]. 

We found that the subunit GINS51 of the GINS complex interacts with the exonuclease subunit of the DNA polymerase D (PolD) ; the interaction between the polymerase subunit of PolD and GINS51 was also detected, albeit displaying higher SR scores (SR-:0,22 ; SR+:0,41). Although this interaction has not been biochemically characterized, it has been reported in the study of Li et al. that GINS51 interacts with both subunits of PolD (Table 2). These congruent data suggest that the interaction between GINS51 and PolD is specific and reinforce the hypothesis that “GINS functions as the center of the replisome, linking the polymerase, helicase, and primase components” [14]. 

**Figure 2:**
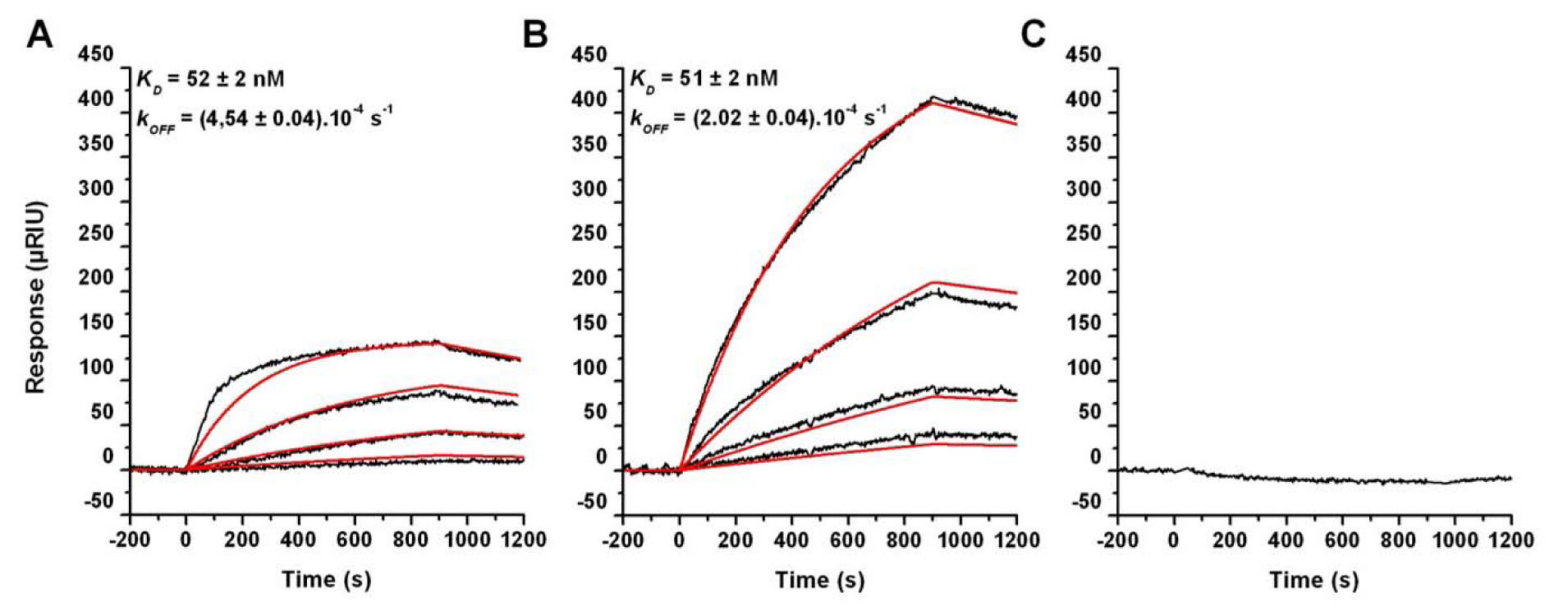
RPA, bound to *ss*DNA, interacts with DNA polymerase D and DNA primase. The catalytic subunit of the DNA primase (**A**), PolD (**B**) and PolB (**C**) were injected at different concentrations (16.67 nM, 50 nM, 150 nM and 450 nM) on a chip coated with the assembly of a 32mer *ss*DNA bound by RPA. Signals reported (black curves) are already subtracted from a RPA dissociation curve and data were fitted using a 1:1 binding reaction model (red curves).

Subunits RPA32 and RPA41 of the RPA complex were co-purified with the HIS-tagged PolD complex. The interaction between RPA41 and PolD was also reported in *Pyrococcus furiosus* using immunoprecipitation experiments [36], suggesting that a specific interaction between RPA and PolD is likely to occur at the replication fork in *P. abyssi*. In this latter report, Komori et al. also reported interactions between RPA41 and both DNA primase and DNA polymerase B (PolB), which were not detected in our high confidence dataset, although His-tagged RPA complex co-isolated both subunits of the DNA primase, but with moderate SR scores (SR-/SR+: 0,09/0,26 for P46 and SR-/SR+:0,36/0,51 for P41). To clarify this situation, we sought to characterize the physical interactions between the RPA complex and the DNA polymerases from *P. abyssi*. To this aim, we designed a SPR experiment using RPA complex immobilized on a 32mer single stranded DNA oligonucleotide to monitor the interaction with the polymerases injected over the flow cell. This experimental set up not only allowed us to orient the RPA molecules on the chip, which is known to improve the analysis of the SPR signals, but also had the potential advantage of approaching the physiological conditions required for these interactions to take place. Figure 2 clearly demonstrated that both PolD and the small catalytic subunit of DNA primase interacted with RPA, bound to ss DNA, with similar affinities, whereas PolB did not bind to RPA complex. According to the dissociation constant measured (Figure 2), the RPA/PolD complex is roughly twice as stable than the RPA/catalytic subunit of DNA primase complex, which could explain the differences observed between the SR scores of PolD and DNA primase in our AP/MS experiments and further indicates that this procedure preferentially reveals stable interactions and is not suited to detect less stable or transient interactions. These SPR results are thus in good agreement with the results of the AP/MS experiments. On the other hand, it is in contradiction with the data of Komori et al. that showed an interaction between RPA and PolB, however, it is not unreasonable to suggest that the very weak signal obtained using immunoprecipitation for PolB in the latter study, when compared to those for DNA primase and PoD, could be an artefact. It has been proposed, based on biochemical properties, that both PolD and PolB from *P. abyssi* could be present at the replication fork and that PolB might be the candidate to replicate the leading strand while PolD could synthesize the lagging strand [37]. The differences in RPA binding could thus reflect separate mechanisms for the initiation and elongation steps of PolD and DNA primase on lagging strand and PolB on leading strand. 

Altogether, with the limitation that not all known complexes could be detected, possibly because they may not assemble under our growth conditions or because the tag interferes with complex assembly, these data indicate that the filtering process of our data is consistent and that the threshold used to sort out the mass spectrometry data is selective enough to ensure that many biologically relevant interactions involved in DNA transactions can be revealed through this approach. 

Besides these already reported interactions, the network encompassed also either, unknown interactions between documented components of the archaeal genomic maintenance, or potential new components. In the next sections, we will describe these associations with a special emphasis on the main PCNA, RPA and DNA primase clusters detected in the network.

### New interacting partners of the PCNA

#### Mre11/Rad50 Complex

**Figure 3:**
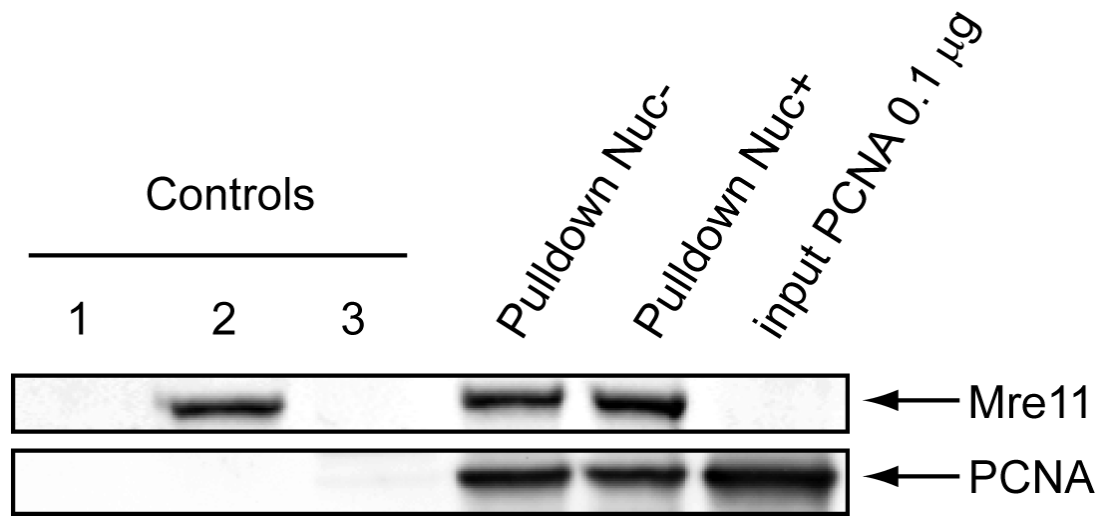
PCNA interacts with Mre11/Rad50 complex. 10 µg of HIS fusion Mre11/Rad50 immobilized on Co2+ magnetic beads were incubated with 0.5 µg of PCNA in absence or presence of DNase I (Pulldown Nuc- or Pulldown Nuc+). Control lanes consisted in the Co2+ beads only (1) or incubated with either the complex Mre11/Rad50 (2) or the PCNA (3). Bait and prey proteins were analysed by Western blot, and probed with PCNA or HIS antibodies.

PCNA, used as bait, co-isolated both Mre11 and Rad50. The reverse experiment, performed with the tagged complex Mre11/Rad50 also co-isolated the PCNA, strongly suggesting that PCNA could form a stable complex with Mre11/Rad50 assemby in *P. abyssi*. PCNA, Mre11 and Rad50 from *P. abyssi* are close homologs to their eucaryal counterparts and as the interaction between these components has not yet been reported in either domain, we sought to confirm this finding. With that in mind, we carried out co-immunoprecipitation *in vitro* with the purified PCNA and Mre11/Rad50 complexes. Figure 3 clearly shows that the Mre11/Rad50 complex co-immunoprecipitated the PCNA in either presence or absence of nuclease treatment, thereby validating the result of the AP/MS experiment. 

The eucaryotic Mre11 complex, comprising of Mre11, Rad50 and Nbs1, is a sensor of double-strand breaks (DSBs) that also controls the DNA damage response by governing the activation of the central transducing kinase ATR/ATM. In addition, the Mre11 complex regulates DSB repair, through the homologous recombination repair (HR) and non-homologous end-joining (NHEJ) [38]. However, a number of recent reports are consistent with the eucaryal Mre11 complex having a primary role in the maintenance of the replication fork during DNA replication and in the HR-dependent resolution of DNA replication-associated DSBs [38]. All the archaeal genomes sequenced to date contain clear homologs of eucaryal Mre11 and Rad50 and the initial biochemical characterization of the *P. furiosus* homologs indicate that the archaeal Mre11 complex is functionally similar to those from Bacteria and Eukarya. However, Nbs1 additional component has not been found in Bacteria and Archaea. In contrast, the HerA helicase and NurA nuclease, specific to the Archaea and frequently found in thermophilic archaeon, have been found to be physically and functionally associated with the archaeal Mre11 complex [39,40,41,42]. Indeed, these four proteins co-operate for the 5’ strand resection at DSB prior to HR [41]. However, the physiological functions of the Mre11 complex remain unsolved in Archaea, due to the conflicting conclusions of the genetic studies performed so far [13,43].

To date, the physical interaction between PCNA and Mre11 complex has never been reported either in *Eucarya* or in Archaea. However, it has been shown that the eucaryotic Mre11 complex co-localizes with the PCNA at replication foci during S phase and that the eucaryal Mre11 complex plays a primary role in the maintenance of the replication fork during DNA replication [44]. If this were also true for the Archaea, it would suggest that the archaeal PCNA could be involved in the recruitment and dynamics of the Mre11 complex at the replication fork, either to stabilize the components of the replisome or to repair stalled replication fork intermediates by HR. Another non-exclusive hypothesis could be that PCNA might also enhance, in some way, the mechanism of end resection carried out by the Mre11 complex, NurA and HerA, prior to strand invasion at DSB sites. 

#### Pab0431, a new nuclease associated with PCNA?

**Figure 4:**
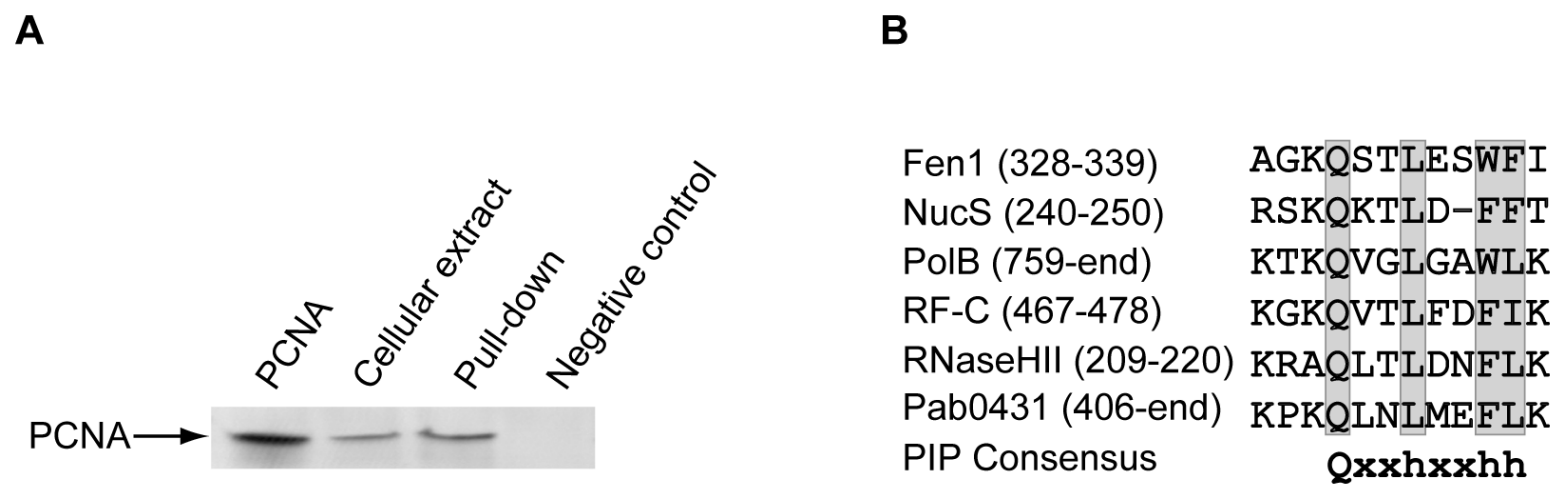
Pab0431 interacts with PCNA and displays a putative C-terminal PIP motif. **A**, 5 μg of histidine-tagged Pab0431 bound to magnetic beads (lane pull-down), and a negative control that consists of the beads only, were incubated with 16 mg of *P. abyssi* cellular extract. Control lanes contained 0.33 μg of cell free extract and 8.75 ng of PCNA. The presence of PCNA on the blot was revealed using anti-PCNA antibody. **B**, Pab0431 has a canonical C-terminal PIP box motif when compared to the consensus sequence (x and h respectively refer to any amino acids and hydrophobic residues) and various sequences of proteins from *P. abyssi* known to interact with the PCNA through the PIP motif. The position of the PIP box within the sequences is indicated.

Pab0431 (Q9V0104 in Figure 1B) co-precipitated with the PCNA bait and we confirmed, using pull-down immunoprecipitation (Figure 4A) that this interaction was specific. Numerous client proteins of the PCNA interact with the replication clamp via a PIP box [24,35,45], a structure which is shown to be well conserved in the C-terminal end of the Pab0431 sequence (Figure 4B). 

Pab0431 possesses a N-terminal PHP (polymerase and histidinol phosphatase) domain (5-226) and a Zn ribbon motif (248-270); it belongs to COG1379, a grouping of proteins found in both Bacteria and the Euryarchaeal phylum of the Archaea (with the exception of the halobacteriales, thermoplasmatales and archaeoglobales orders), which have no clear function. However, the PHP domain is a putative phosphoesterase domain found, isolated or associated, in proteins with diverse functions, including bacterial DNA polymerase III, eucaryotic DNA polymerase, X-family of DNA polymerases and histidinol phosphatases [46]. This domain consists of four conserved core regions containing invariant histidine and aspartate residues implicated in metal ion coordination. These four motifs were found conserved in the sequence of Pab0431 (data not shown).

 An HHpred search (http://toolkit.tuebingen.mpg.de/hhpred) with the pab0431 sequence indicated significant similarity (E-value=2.2e-61) with the N-terminal sequence (7-436) of DNA polymerase III alpha subunit (PolIIIα) from *Thermus aquaticus*. This region contains a PHP domain which is present in all PolIIIα sequences and was found to exhibit zinc ion-dependent 3’-5’ exonuclease activity [47]. Pab0431 also displays similarity to a stand-alone PHP domain of the YcdX protein from *E. coli* that is closely related to the PolIIIα PHP domain. The structure of YcdX revealed a Zn2+ trinuclear center with characteristics similar to several phosphoesterases [48]. Interestingly, YcdX is significantly induced in response to DNA damage caused by mitomycin C in *E. coli* [49]. 

We demonstrated that Pab0431 is a new partner of the PCNA and is thus likely to be involved in a DNA metabolism process. Pab0431 displays a canonical PIP motif at its C-terminal end and although mutation analyses are needed to assess the role of this motif regarding the interaction detected, it is likely that this sequence could mediate the interaction with the replication clamp. The presence of a PHP domain, containing the four conserved motifs responsible for metal coordination, as well as a Zn-ribbon motif tips the balance in favour of potential metallo-dependent hydrolase and nucleic acid binding activities. In addition, Pab0431 has significant similarities to the PHP domain of bacterial PolIIIα, recently described to have a zinc dependent proofreading activity, and with the YcdX protein of *E. coli* that contains a stand-alone PHP domain and also may be related to DNA repair. Taken together, these data suggest that Pab0431 may have a nuclease activity related to DNA replication, as a separate subunit providing an additional proofreading activity for the replicative DNA polymerases, or could be involved in a DNA repair process that remain to be determined. This ‘hypothetical protein’ thus appears to be an exciting target for further functional characterization. 

### RPA serves as a mediator for genome maintenance in *P. abyssi*

#### RPA activates transcription in vitro

**Figure 5:**
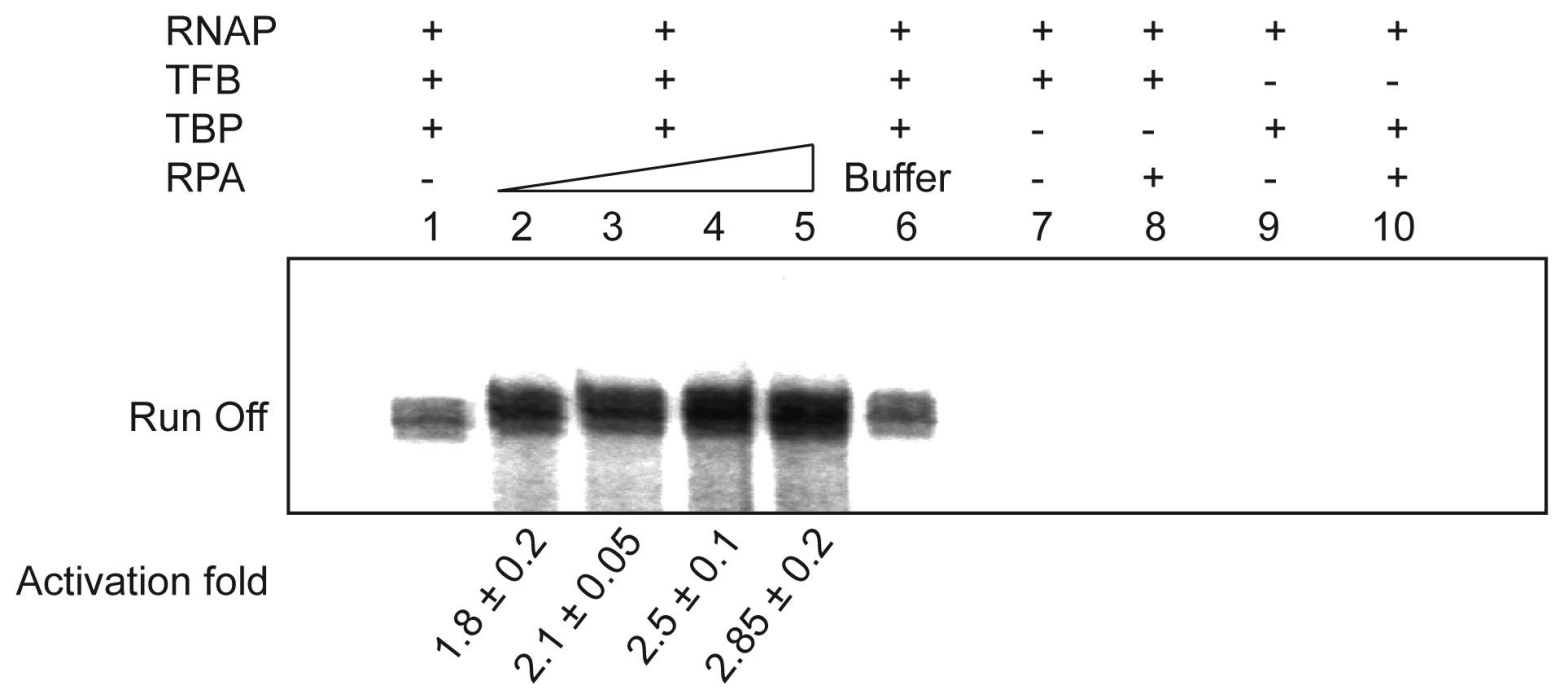
RPA stimulates transcription *in* *vitro*. The effect of RPA on transcription was analysed by *in* *vitro* transcription experiments either in the absence or in the presence of increasing amounts of RPA (10 nM, lane 2; 20 nM, lane 3; 30 nM, lane 4; 50 nM, lane 5). The presence or absence of TBP or TFB is indicated on top of the lanes. The effect of RPA on transcription activity from three replicates was quantified by phosphorimaging (Fujifilm FLA-5000) and indicated as activation fold, with standard deviation, at the bottom of each lane.

The interaction observed between the large subunit RPA41 of the heterotrimeric RPA complex and the two A1 and A2 subunits of the RNA polymerase (RNAP) suggested the possibility that RPA could play a role in transcription (Figure 1B). As the purified RNAP from *P. abyssi* was not available, we tested this hypothesis using the basal transcription machinery from *P. furiosus* and the RPA complex from *P. abyssi* in a promoter-specific transcription assay using the *gdh* promoter as a template [26]. Both RPA and RNAP complexes from these two *Pyrococcus* strains display high sequence identities and the numbers and domain organisation of the subunits are similar [36,50]. RPA complex was incubated with DNA, Transcription Factor B (TFB) and TATA-box Binding Protein (TBP). Reactions were initiated by addition of nucleoside triphosphates and the corresponding run-off transcript was formed (Figure 5). Interestingly, with the addition of increasing amounts of RPA the total amount of synthesized transcripts also accumulated up to almost three fold (Figure 5, compare lanes 1-6). Control experiments with buffer or in the absence of transcription factors confirmed that the activation of transcription depends on the presence of RPA. The same transcription profile was observed with the circular template (*gdh* template cloned in pUC19 ; data not shown).

Archaea have been found to possess multiple RPA and SSB homologs and among the euryarchaea, RPAs display an unusual diversity and distribution [30,51]. The RPAs from *P. furiosus* and *P. abyssi* exist as a stable hetero-oligomeric complex consisting of three subunits, RPA41, RPA14, and RPA32 [36]. Comparisons of amino acid sequences and protein domain organisations strongly suggest that the euryarchaeal RPA proteins are more closely related to the eucaryal one than to the crenarchaeal and eubacterial SSBs. Studies of archaeal RPAs and SSBs have provided many insights into the role these single strand DNA binding proteins serve in DNA processing pathways, including DNA replication, recombination and repair. In particular, the crenarchaeal SSB of *Sulfolobus solfataricus* was also shown to enhance transcription in TBP limiting conditions [52], but this is the first study demonstrating that a Euryarchaeal heterotrimeric RPA, reminiscent to the organisation of eucaryotic RPA, increases transcription *in vitro*.

Archaea and Eukarya share a common basal transcription apparatus that consists of a multisubunit RNAP and three transcription factors, TBP, TFB, and transcription factor E. Richard et al. reported a specific physical interaction between the SSB and RNAP from *S. solfataricus* and demonstrated that SSB supports transcription in the absence of TBP and stimulates the polymerase at the level of recruitment and/or initiation [52]. They suggested a model in which SSB in transcription could have a role in disruption of chromatin structure at the promoter and recruitment of RNA polymerase to form the pre-initiation complex. In sharp contrast with this model, using abortive transcription experiments, we demonstrated that archaeal RPA did not stimulate initiation of transcription and could not substitute for a basic transcription factor in initiation (full analysis to be presented elsewhere). These properties were rather indicative of a mechanism of stimulation of transcription similar to the one demonstrated recently for the eucaryal RPA heterotrimer. Indeed, quantitative proteomic analyses highlighted the yeast RPA complex as an interaction partner of the transcription machinery [53]. Further genetic and biochemical experiments led to the suggestion that RPA interacts with the non-template strand of RNA polymerase II complexes during elongation. However, the function of eucaryal RPA associated to the elongating-transcription complex is still unknown. In that regard, further works are needed to determine whether the archaeal RPA may also interact with elongation complexes and bind to non-transcribed templates. Further understanding of the functional and physical interaction found between archaeal RPA and RNAP could provide exciting advances on transcription mechanisms both in Archaea and *Eucarya*.

#### Is there a Dna2-like dependent pathway for Okazaki fragments maturation in *Archaea* ?

An association was found between the subunit RPA32 and the protein Dna2-like helicase (Pab0067) using either the RPA complex or Dna2-like homolog as bait, strongly suggesting that this interaction is specific. As the full Dna2-like protein from *P. abyssi* was poorly expressed and essentially, in the insoluble fraction, we truncated the protein into two domains, the N-terminal portion encompassing the predicted nuclease domain of RecB family (1-600) and the C-terminal halves comprising the superfamily I DNA/RNA helicase domain (601-1308). We only succeeded to express and purify the C-terminal helicase domain and used this truncated domain as bait in our AP/MS assay, thereby suggesting that this portion of Dna2-like potentially interacts with RPA. Pab0067 encodes a distantly related homologue of the eucaryal Dna2 protein, which is formed by the fusion of a helicase and an endonuclease domain. Dna2 is postulated to cleave long DNA flaps that escape Fen1 activity during Okazaki fragment (OF) maturation in yeast [54]. In contrast, human Dna2 seems to play a role in DNA replication that is distinct from Fen1 and OF maturation [55]. It has already been demonstrated that OF have similar sizes in *P. abyssi* and *Eucarya* (around 150 nucleotides) [56]. A recent biochemical study has suggested that the coordinated activities of RNaseHII, Fen1, DNA polymerases B and D and DNA ligase1 are involved in RNA primer removal in *P. abyssi* [57]. This study was performed using short flap substrates (max 16 nt), thereby not precluding the possibility that another pathway might also exist to cleave long DNA flaps that escape Fen1 activity. In this regard, the interaction between RPA and Dna2-like could be indicative of an alternative RPA/Dna2-like/Fen1 pathway for maturation of OF in Archaea.

### The DNA primase clusters with RadA and DNA ligase in the network

Interestingly, the primase cluster consisted of the catalytic subunit of DNA primase, DNA ligase and RadA, suggesting that DNA primase and DNA ligase I could be implicated in recombinational repair of DNA double-strand breaks. Given the relative paucity of DNA polymerases dedicated to repair functions in archaeal genomes and the architectural connection between Pol X DNA polymerases and the small subunit of archaeal primases [58], it is not unreasonable to propose that the archaeal DNA primase could also have DNA repair functions. In this respect, the primase complex from *S. solfataricus* was shown to be capable of template-dependent polymerization across discontinuous DNA templates [59]. Based on this new biochemical property, it was suggested that DNA primase may be involved in double-strand break repair in Archaea, which is consistent with our data.

Independently of the clusters detected, other associations in the network attracted our attention and, we believe, provide firm basis for further investigations.

### Is XPD an accessory helicase at the replisome?

Strikingly, the helicase XPD was found in interaction with the supposed replicative helicase MCM, independently of the main network (Figure 1). The presence of XPD only in the MCM pull-down and its total absence in the five independent negative controls, strongly suggests that we are observing a true interaction, rather than a non-specific effect. 

XPD is a superfamily-2 (SF2) 5'-3' helicase containing an iron-sulphur cluster [60]. As a component of transcription factor II H (TFIIH), the eucaryal XPD is involved in DNA unwinding during nucleotide excision repair (NER) [61]. Archaeal XPD is closely related in sequence to the eucaryal enzyme but the biological functions of XPD in archaeal cells still remain unclear. Biochemical properties are consistent with an implication in DNA excision repair pathway [62] but a recent genetic analysis of DNA repair in *T. kodakarensis*, suggested that XPD does not seem to play a major role in *T. kodakarensis* NER pathway, given the high level of resistance to UV, methyl methanesulfonate and mytomycine C exhibited by the *Δxpd* mutant [13]. Moreover, apart from our study, no physical interactions between archaeal XPD and other proteins have been demonstrated that could give clues to the physiological functions of this helicase or enforce the hypothesis of a role in archaeal NER. 

A recent genetic study demonstrating that DinG is essential for efficient replication across highly transcribed regions in *E. Coli* [63] may shed light on the interaction found between MCM and XPD. Indeed, DinG is a bacterial representative of the SF2 family of helicases and translocates in the 5'-3' direction on ssDNA. The bacterial enzyme behaves *in vitro* similarly to the archaeal XPD [64] and knockouts do not render *E. coli* sensitive to DNA damaging agents [65]. Using construct strains carrying chromosomal inversions, Boubakri and co-workers, demonstrated that DinG is recruited to the replication fork to allow replication across oppositely oriented transcribed ribosomal operons, removing R-loops and participating in RNA polymerase removal [63]. 

In Archaea, the enzymes implicated in clearing away the path of the replication fork from the RNA polymerase are not known, however, it has been shown *in vitro* that the replicative helicase MCM of *Methanothermobacter thermautotrophicus* could not unwind DNA in the presence of stalled transcription elongation complex, which raised the hypothesis that, at least *in vitro*, additional proteins may facilitate the removal the RNA polymerase complex [66]. In this light, and without ruling out a possible role of XPD in NER pathway, it raises the hypothesis that this helicase could also be implicated in RNA polymerase removal. In this respect, after fork arrest due to collisions between replication and transcription complexes, archaeal XPD could be recruited and then act either alone or in a concerted manner with MCM to dislodge the RNA polymerase and remove the R-loops. Another possibility could be that XPD travels with the fork and may act in preventing fork arrest and separation of the replisome components upon collisions. The activities of archaeal XPD against R-loops and DNA bound by the RNA polymerase has not yet been tested, further genetic and biochemical experiments are thus required to confirm this hypothesis.

### Pab0961 potentially couple DNA replication and DNA recombination at the replication fork

Pab0961 was found associated with RadB and a NAD-dependent protein deacylase of Sir2 family (Pab0801) in the high confidence dataset of proteins (Table S1). 

Pab0961 is a member of the CBS-domain-containing membrane protein COG3448. This protein is misannotated as an Inosine-5'-monophosphate dehydrogenase, but in fact it is not an enzyme and contains only two non-catalytic pairs of Cystathionine β-synthase (CBS) domains. These domains are often found in proteins with completely different functions, which contain other domains that are usually enzymatic, membrane transporters or DNA-binding domains. It has been shown that CBS domains regulate enzyme activity based on the concentration of AMP/ATP or other adenosine derivatives, but they may also bind metallic ions such as Mg^2+^ [67,68]. Pab0961 does not contain additional domains and is thus likely devoid of enzymatic activity. Standalone CBS domain proteins, found particularly in prokaryotes, might form complexes upon binding to other proteins to which they interact with and regulate. 

Interestingly, when we weighted the SR ratio of the MS results with operons and genomic neighbourhood data (Archaeal Genome Browser [69]), GINS23, which is encoded in close vicinity and pulled down Pab0961 with a moderate SR score (SR- :0.23 ; SR+ :0.27), was reclassified as a putative interacting partner of this protein. The interaction between GINS23 and TK1186, the homologue of Pab0961 in *T. Kodakarensis*, was also observed by Li and co-workers [14], indicating that this interaction is likely specific. In addition the recently described GINS associated nuclease (GAN) of the RecJ family, used as bait in our AP/MS assay, also co-purified Pab0961, but only in the nuclease free assay. GINS, GAN and MCM form a complex that appears to be a key component of the DNA replication machinery in all Archaea [17]. This complex is homologous and most likely functionally analogous to the eucaryotic CMG complex. 

The fact that this standalone CBS domain protein co-purified with GINS23 subunit in two independent studies and at least in the nuclease free assay with the GAN nuclease raises the hypothesis that Pab0961 might be involved in the regulation of the activity of the archaeal CMG complex. In addition, the interaction detected with RadB, raised also the hypothesis that this protein could link DNA replication and DNA recombination at the replication Fork. In support to this hypothesis, the physical interaction between the homologs of Pab0961 (Hvo_2384) and both RadA and RadB has been demonstrated *in vivo*, in *Haloferax volcanii* (Thorsten Allers, personal communication). 

It has been proposed that prokaryotic stand-alone CBS domain proteins interact with and regulate the activities of effector proteins by binding adenine nucleotides depending on the energy charge of cells [70]. Given the central roles of CBS domains in eucaryotic metabolism, it is thus likely that they also play important roles in the cellular physiology of prokaryotes.

A major contribution of the present study is that we reveal for the first time the identity of such potential effectors proteins presumably linking DNA replication and DNA recombination at the replication fork, the activity of which could be regulated in response to the energy charge of cells. Further genetic and biochemical experiments are now required to better understand the function of this new regulation protein.

## Conclusions

In this report we describe the first protein interaction network of genomic maintenance in Archaea and provide experimental confirmation for some of the associations detected. Although this study did not allow us to solve the mystery of how HA preserve genome integrity in such a harsh environment, it provides valuable information on novel molecular associations for which elucidating the function might reveal new mechanisms in DNA pathways. We also discovered new potential actors exhibiting both fundamental and biotechnological interest, as exemplified by the protein Pab0431 of unknown function, which we propose to be a new nuclease associated with the PCNA.

In particular, we observed interactions between replisome and DNA recombination components, PCNA-Mre11/Rad50, DNA primase-RadA, DNA ligase-RadB. One simple explanation could be that these associations suggest that replication can initiate from recombination intermediates, possibly because, recombination intermediates can be generated from archaeal replication forks. Thus, these results seem to indicate a close coordination of DNA replication and recombination activities in the maintenance of genome stability of hyperthermophilic Archaea. HA might have evolved a high-fidelity recombination-dependent replication mechanism that can accurately repair the damage, which could thereby explain the lack of translesional polymerases observed in euryarchaea.

Indeed, in organisms where replication starts from a single origin (or at least few origins), restarting mechanisms assist fork progression by exploiting the homologous recombination DNA repair machinery. In Archaea, both RadA, the genuine recombinase and RadB, a recombination mediator, are required during normal growth [71], thereby indicating that this requirement during growth is likely related to replication restart. In this context, the interactions between components of the replication and recombination machineries might suggest that a break-induced replication mechanism is active in Archaea after fork collapse when a replication fork encounters nicked DNA.

 This interplay between the recombination and replication machineries likely interfaces with regulatory elements involved in the control of the DNA damage response, as exemplified by the identification of a new factor, Pab0961, presumably involved in the coupling of DNA recombination and DNA synthesis at the replication fork. 

We hope that this promising initial study will provide the cornerstone for a deeper understanding of how the cellular processes of genomic maintenance are coordinated in Archaea. Further functional studies are now fundamental to unravel the complexities of these interactions with the potential to highlight the molecular mechanisms implicated in similar processes in Eucaryotes.

## Supporting Information

Figure S1 **Level of purification of the tagged-proteins or domains produced in *E. coli*.** A sample (1-3 µg) of the affinity purified baits, used in this study, was loaded on a SDS precast gels (Criterion XT, Biorad). The arrows indicate the position of the histidine-tagged baits and the asterisk, the position of the 50 kDa band from the molecular weight marker (MW).
(TIF)

Materials S1 **Additional materials and methods.**
(DOCX)

Table S1 **High confidence subset of the interactions detected.**
(PDF)
