# Supplementary figures and images for: An Extended Network of Genomic Maintenance in the Archaeon Pyrococcus abyssi Highlights Unexpected Associations between Eucaryotic Homologs
Source: PLoS One. 2013 Nov 7;8(11):e79707. doi: 10.1371/journal.pone.0079707 (PMC3820547; doi:10.1371/journal.pone.0079707)

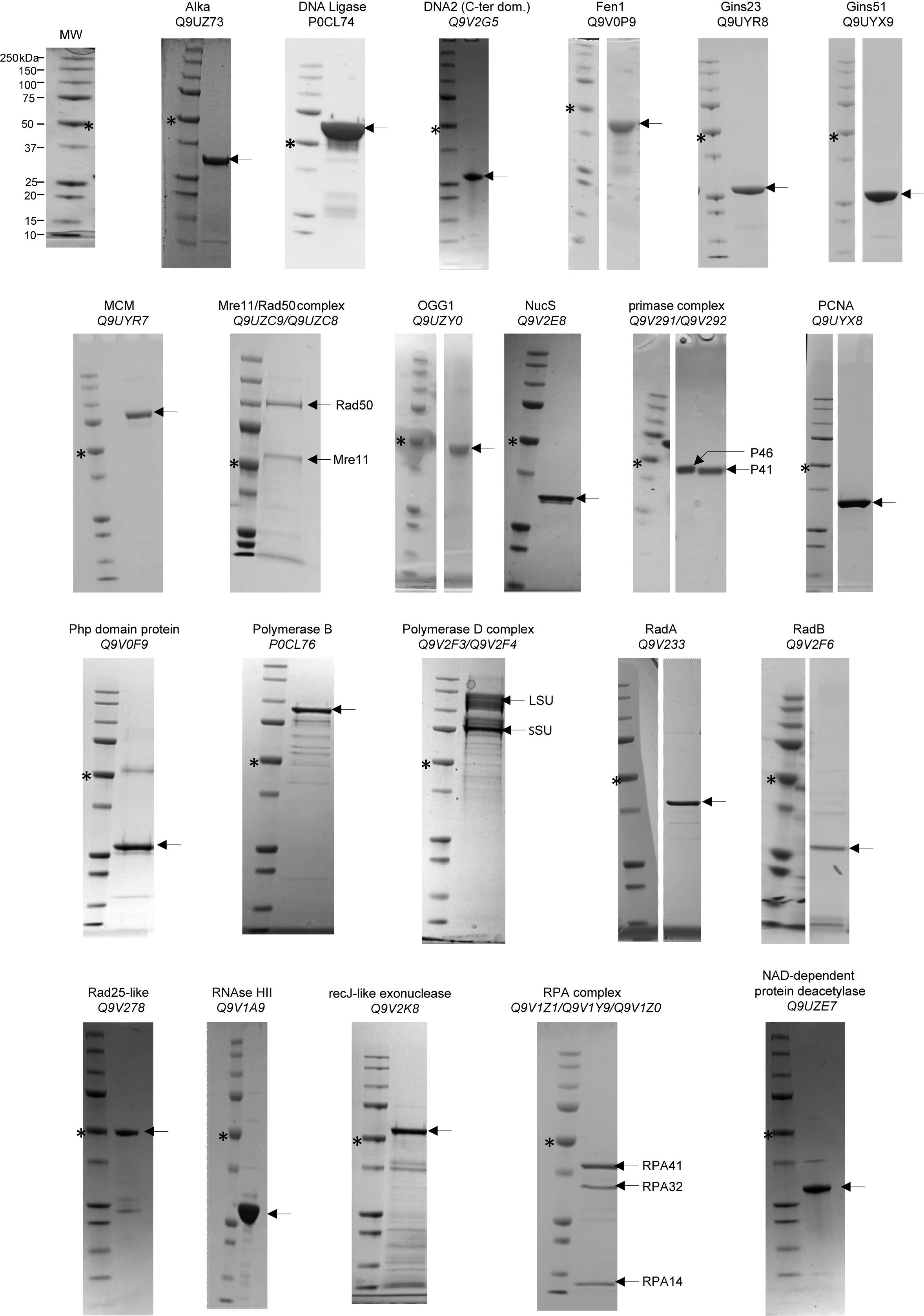

Supplement: Figure S1 — Level of purification of the tagged-proteins or domains produced in E. coli. A sample (1-3 µg) of the affinity purified baits, used in this study, was loaded on a SDS precast gels (Criterion XT, Biorad). The arrows indicate the position of the histidine-tagged baits and the asterisk, the position of the 50 kDa band from the molecular weight marker (MW). (TIF) [file pone.0079707.s001.tif]
