# Supplementary material for: An Extended Network of Genomic Maintenance in the Archaeon Pyrococcus abyssi Highlights Unexpected Associations between Eucaryotic Homologs
Source: PLoS One. 2013 Nov 7;8(11):e79707. doi: 10.1371/journal.pone.0079707 (PMC3820547; doi:10.1371/journal.pone.0079707)
Supplement: Materials S1 — Additional materials and methods. (DOCX) [file pone.0079707.s002.docx]

**MATERIAL AND METHODS**

**Cloning, production and purification of the RPA complex.**

The structural genes of *Pab*RPA41, *Pab*RPA14 and *Pab*RPA32 were amplified using the genome of *Pyrococcus abyssi* as a template, the high fidelity Pfu DNA polymerase (Promega) and the following primers:

RPA41_For: 5’-CAAAGGTGGTTCATATGACGGTGTTGACG-3’

RPA41_Rev: 5’-CTCTAACTTGAGGATCCATCTCACATCATC-5’

RPA14_For: 5’-GGGTGATGTCATATGGAAGTTCAAGTTAGG-3’

RPA14_Ter: 5’-CCTAGTCGCGGGGATCCTCTTCTTCATTTC-3’

RPA32_For: 5’-GTGTGGTGATTCATATGAAGAAGAGGATGC-3’

RPA32_Rev: 5’-CCTTCCATGGGATCCACCTTACCCTCTATC-3’

The PCR products were digested by *Nde*I and *BamH*I and cloned into the pET 28a+ vector (Novagen) for RPA41 and into the pET 25b+ vector (Novagen) for RPA14 and RPA32. Thereafter, to improve the solubility of RPA32, the gene cloning for this subunit was inserted in frame with the gene of RPA14 and under the T7 promoter control. To this aim, the recombinant pET 25b+/RPA14 and pET 25b+/RPA32 plasmids were double digested by *BamH*I/*Not*I and *Bgl*II/*Not*I, respectively. The *Bgl*II-*Not*I fragment carrying the RPA32 and the T7 promoter as well as the ribosome binding site sequence was then ligated with the *BamH*I-*Not*I fragment, hence downstream and in frame with the RPA14 gene. The resulting plasmids, pET 28a+/RPA41 and pET 25b+/RPA14-RPA32, were used to transform *Escherichia coli* Rosetta (DE3) pLysS strain (Novagen) for the production of the three subunits, with RPA41 fused to a histidine tag at the N-terminus. The nucleotide sequence of each plasmid was checked by DNA sequencing. Although numerous clones have been tested, the insert corresponding to the gene of RPA41 was always found with at least one mutation in the 5’ part. One of these mutated clones exhibited a transition leading to a leucine 4 to valine mutation of the amino acid deduced sequence. The attempts to correct this mutation failed and induced additional mutations; consequently, as this mutation was considered to be conservative enough, the clone was used for further analysis. Cells were grown in 8 litres of LB medium supplemented with chloramphenicol (34 μg/ml), ampicillin (50 μg/ml) and kanamicin (50 μg/ml) at 37°C to A_600nm_ = 0.6 and expression of RPA proteins were induced by the addition of 0.5 mM IPTG (final concentration). Four hours after induction, cells were harvested by centrifugation and stored at -80°C. The pellets were resuspended in 100 ml of lysis buffer (30 mM Tris-HCl, pH 7.5, NaCl 300 mM, ZnCl_2_ 10 µM and imidazol 10 mM). Cells were heated at 80°C for 15 minutes and then frozen at -80°C for 30 minutes; this freeze/heat cycle was applied three times. Cell debris were removed by centrifugation (12 000 g for 30 minutes at 15°C). Most of the remaining *Escherichia coli* proteins were removed using another heat step: 30 minutes at 80°C, followed by a centrifugation step (12 000 g for 30 minutes at 15°C). Supernatant was filtered (0,40 µm) and loaded onto a Ni^2+^ affinity column pre-equilibrated in buffer A (30 mM Tris-HCl, pH 7.5, NaCl 300 mM and imidazol 10 mM). Non-bounded fraction, containing the small subunit, was collected, filtered using a 30kDa-cutoff concentrator (Vivaspin, Sartorius), heated (80°C for 15 minutes) and centrifugated (12 000 g for 30 minutes at 15°C). A gradient of buffer A to buffer B (30 mM Tris-HCl, pH 7.5, NaCl 300 mM and imidazol 500 mM) was applied to the column, and the His tagged RPA complex was eluted at 100 mM imidazol. Fractions containing the heterotrimer RPA were pooled and concentrated to 10 mg/ml, and applied onto a size-exclusion column (Superdex 200 – GE Healthcare) equilibrated in buffer C (30 mM Tris-HCl, pH 7.5, NaCl 300 mM). Excess of medium subunit appeared to be adsorbed on the column, and was released using NaOH 200 mM. Fractions were titrated with 1 M Tris-HCl, pH 7.5 and protein was stored in buffer C. To eliminate DNA in each protein or complex samples, NaCl concentration was adjusted to 1 M and Poly-imine P was added to a final concentration of 0,5%. DNA was then removed by centrifugation (15 000 g for 1 hour at 15°C). Protein was precipitated with ammonium sulphate (sigma) to remove Poly-imine P, and solubilized in buffer D (30 mM Tris-HCl, pH 7.5, NaCl 500 mM). Ammonium sulfate was eliminated by dialysis against buffer D. The large subunit (RPA41) and the full complex were then separated on an size-exclusion column (Superdex 200 – GE Healthcare) equilibrated in the same buffer. Fractions containing subunits alone or the complex were separately pooled and concentrated to 10 mg/ml, and stored at 12°C in buffer D.

**Cloning, production and purification of MCM.**

The *mcm* gene, was shown to contain two intein coding regions, which are intervening sequences spliced out as a protein and not as a mRNA. To prevent a possible toxic effect of the inteins in *E. coli*, the genes encoding inteins were deleted using the splicing by overlap extension PCR approach [1], with the following primers:

MCM-ini: 5’-GGATGGGTGAAGCATATGGATAGAGAGGAG-3’

MCM-int1R: 5’-ATGTATCTGAGAAGTTGGCTGTTTTTCGCGACTCCCGGATCTC-3’

MCM-int1F: 5’-GAGATCCGGGAGTCGCGAAAAACAGCCAACTTCTCAGATACAT-3’

MCM-int2F: 5’-TCGTCCCTAACCACCGCGGCGAGCCCAGCGGCAGAACTGC-3’

MCM-int2R: 5’- GCAGTTCTGCCGCTGGGCTCGCCGCGGTGGTTAGGGACGA-3’

MCM-end: 5’-AAAGAGTAGTCGACTCAGACGGTTCTGTAATA-3’

The resulting PCR product contained the MCM gene minus the intein. This product was double digested by *Nde*I/*Sal*I (restriction sites in bold in the sequences of primers), cloned into pQE-80 and sequenced. The constructed expression vector pQE-80L/*Pab*MCM was introduced into host *E.coli* Rosetta (DE3) pLysS. The transformed cells were grown at 37°C in 6 L LB medium supplemented with 50 µg/ml ampicillin and 34 µg/ml chloramphenicol. When culture reached an A_600_ of 0.6 OD, protein expression was induced by addition of 1 mM IPTG. Induced cells were incubated at 37°C during 4 hours and harvested by centrifugation. The pellet was resuspended in lysis buffer (10 mM Tris HCl
pH 7.5, 150 mM NaCl, 1 mM DTT, and Complete Mini-ethylenediaminetetraacetic acid [EDTA]-free anti protease (Roche)). Cells lysis was done using "One Shot" Cell Disrupter (Constant Systems LDT, Daventry, UK) at a pressure of 1.9 kbars. The resulting lysate was centrifuged at 8000 x g for 15 min at 4°C to eliminate cell debris. The supernatant was then subjected to heat treatment at 75°C for 10 min and the thermo-precipitated proteins were removed by centrifugation at 8000 x g for 15 min at 4°C. The clarified supernatant was applied to a HisPrep FF 16/10 column (GE Healthcare Life Sciences) preequilibrated with buffer A (10 mM Tris HCl pH 7.5, 150 mM NaCl, 10 mM imidazole, 1 mM DTT). Elution was performed with 140 ml of 0.01 to 0.5 M imidazole linear gradient in buffer A. 1.0 ml fractions were collected and analyzed by SDS-PAGE to detect the MCM polypeptide. Fractions containing MCM were pooled and dialyzed overnight against buffer B (10 mM Tris HCl pH 7.5, 150 mM NaCl, 1 mM DTT). The dialysate was loaded onto a Superdex S200 10/300 GL column (GE Healthcare Life Sciences) pre-equilibrated with buffer B. The peak fractions were pooled and dialyzed against buffer B and concentrated using a Vivaspin Turbo 15 concentrator (Sartorius) at 2.7 mg/ml. The purified enzyme was then aliquoted and stored at −20 °C.

**Cloning, production and purification of the Mre11/Rad50 complex**

The genes encoding *Pab*0810 (*Pab*Mre11) and *Pab*0811 (*Pab*Rad50)  have been amplified targeting *P. abyssi* genomic DNA with high fidelity Pfu DNA polymerase (Promega) and the following primers:
Mre11_Lic_For: 5'-GACGACGACAAGATGTGGACTTCTGAGGTGTTCG-3 '
Mre11_Lic_Rev: 5'-CGCGGGCGGCCGTCACCCTTTAACCCAGGCC -3 '
Rad50_Lic _Pour: 5'-GCGGGCCCGGCCTGCATGAAGATAGAGGAGC-3 '
Rad50_Lic _rev: 5'-GAGGAGAAGCCCGGTTCATGACACCACCTCTACC -3 '

Both PCR products were inserted in the same pET-30 Ek/LIC Vector (Novagen) that allowed multiple ORF cloning in the same plasmid. *Pab*Mre11 was fused to a HIS-tag at the N-terminus. The recombinant plasmid was used to transform competent *E.coli* BL21(DE3). The transformed cells were grown at 37°C in 6 L LB medium supplemented with 50 µg/ml of kanamycin. When culture reached an A_600_ of 0.6 OD, protein expression was induced by addition of 1 mM IPTG at 15°C overnight. Cells were harvested at 4°C for 45 min at 5000 g. The pellet was resuspended in lysis buffer (20 mM Tris HCl pH 8, 20 mM Imidazole, 1 mM DTT, and Complete Mini-ethylenediaminetetraacetic acid [EDTA]-free anti protease (Roche). Cells lysis was achieved after three runs in French pressure cell (40K cell, 1000 psi). The resulting lysate was centrifuged at 10000 x g for 15 min at 4°C to eliminate cell debris. The supernatant was then subjected to heat treatment at 75°C for 10 min and the thermo-precipitated proteins were removed by centrifugation at 14000 x g for 45 min at 4°C. The clarified supernatant was filtered (0,40 µm) and loaded onto a Ni^2+^ affinity column pre-equilibrated in buffer A (20 mM Tris-HCl, pH 8, 20 mM Imidazol, 1 mM DTT). A gradient of buffer A to buffer B (20 mM Tris-HCl, pH 8, 1 M Imidazol, 150mM NaCl, 1 mM DTT) was applied to the column, and the His-tagged Mre11/Rad50 complex was eluted at 400 mM imidazol. Fractions containing the heterodimer were pooled, dialyzed overnight against buffer C (20 mM Tris-HCl, pH 8, 150mM NaCl, 1 mM DTT) and concentrated using a Vivaspin Turbo 15 concentrator (Sartorius) at 0.3 mg/ml. The purified complex was then aliquoted and stored at −20 °C.

**REFERENCES**

1. Ho SN, Pullen JK, Horton RM, Hunt HD, Pease LR (1990) DNA and protein engineering using the polymerase chain reaction: splicing by overlap extension. DNA and Protein Engineering Techniques 2: 50-55.
