# Supplementary material for: An Extended Network of Genomic Maintenance in the Archaeon Pyrococcus abyssi Highlights Unexpected Associations between Eucaryotic Homologs
Source: PLoS One. 2013 Nov 7;8(11):e79707. doi: 10.1371/journal.pone.0079707 (PMC3820547; doi:10.1371/journal.pone.0079707)
Supplement: Table S1 — High confidence subset of the interactions detected. (PDF) [file pone.0079707.s003.pdf]

Table S1: High confidence subset of the interactions detected

Bait	*Prey*	*Annotated function*	*SR-*	*SR+*
**MCM**	Q9UYR7	MCM	0,00	0,00
	Q9UZ12	ERCC2/XPD	0,09	0,12
	Q9V114	RNAP rpoA1	0,12	0,15
**Gins 23**	Q9UYR8	Gins23	0,04	0,04
	Q9V0Z9	Atp-binding transport protein	0,20	0,12
	Q9UYR2	Q9UYR2	0,19	0,16
**Gins 51**	Q9UYX9	Gins51	0,00	0,00
	Q9V2F3	DNA polymerase D ssu	0,07	0,11
**RecJ-like exonuclease**	Q9V2K8	RecJ-like single-stranded exonuclease	0,00	0,00
	Q9V256	Q9V256	0,04	0,20
**PCNA**	Q9V0P9	Fen1	0,03	0,02
	Q9V1Z5	ERCC4/XPF	0,06	0,11
	Q9UYE0	Metallo-beta-lactamase pab1035	0,07	0,10
	P0CL76	DNA polymerase B	0,08	0,06
	Q9V104	Q9V104	0,08	0,05
	Q9V2E8	NucS	0,09	0,07
	Q9V2G3	RFC-ls	0,10	0,16
	Q9UZC8	Rad50	0,11	0,02
	Q9UZC9	Mre11	0,11	0,03
**DNA polymerase B**	P0CL76	DNA polymerase B	0,00	0,01
**DNA polymerase D**	Q9V2F4	DNA polymerase D ls	0,00	0,00
	Q9V2F3	DNA polymerase D ss	0,00	0,00
	Q9V1Z1	RPA32	0,04	0,09
	Q9V291	DNA primase P46	0,06	0,13
	Q9UY07	Nucleotidyltransferase	0,07	0,16
	Q9V1Y9	RPA41	0,10	0,19
**DNA primase P41**	Q9V233	radA	0,01	0,01
	Q9V292	DNA primase P41	0,04	0,02
	P0CL75	DNA ligase	0,10	0,08
**DNA primase P46**	Q9V291	DNA primase P46	0,00	0,00
	Q9V233	radA	0,03	0,03
**DNA Ligase**	P0CL74	DNA Ligase	0,00	0,00
	Q9UZ86	Reverse gyrase	0,09	0,12
	Q9V114	RNAP rpoA1	0,12	0,15
**Fen1**	Q9V0P9	Fen1	0,00	0,00
	Q9UYE0	Metallo-beta-lactamase pab1035	0,09	0,06
	Q9UYX8	PCNA	0,11	0,11
**RNAseHII**	Q9V1A9	Ribonuclease HII	0,00	0,00
	Q9V2M1	putative ATP-dependent RNA helicase	0,16	0,18
**DNA2 homolog**	Q9V2G5	DNA2 homolog	0,01	0,00
	Q9UY85	RecJ-like phosphoesterase	0,04	0,02
	Q9V1Z1	RPA32	0,04	0,02
	Q9UYI3	GTP-binding protein	0,04	0,03
	Q9V0E5	RNA-binding protein	0,08	0,17
	Q9UZB6	DNA helicase, putative	0,09	0,10
	Q9V2F9	RNA-binding protein AU-1	0,10	0,09
	Q9V1V1	50S ribosomal protein L6P	0,11	0,18
	Q9UZ86	Reverse gyrase	0,14	0,12
	Q8J2X9	Q8J2X9	0,14	0,06
	Q9UZN0	7-cyano-7-deazaguanine tRNA-ribosyltransferase	0,08	0,20
**RPA**	Q9UY07	Nucleotidyltransferase	0,00	0,00
	Q9V1Z1	RPA32	0,00	0,00
	Q9V1Y9	RPA41	0,01	0,00
	Q9V1Z0	RPA14	0,01	0,00
	Q9V1Z5	ERCC4/XPF	0,04	0,08
	Q9V113	RNAP rpoA2	0,05	0,08

	Q9UYS8	DNA topoisomerase 1	0,06	0,07
	Q9UZM4	Lhr-2 large helicase-related protein	0,06	0,05
	Q9UZ86	Reverse gyrase	0,06	0,14
	Q9V114	RNAP rpoA1	0,06	0,10
	Q9V0H2	Lhr-1 large helicase-related protein	0,06	0,09
	Q9V2G5	DNA2	0,07	0,10
	Q9UYC4	ERCC3/XPB	0,09	0,13
	Q9V1X8	HsdM	0,10	0,11
**NucS**	Q9V2E8	NucS	0,00	0,00
	Q9UYX8	PCNA	0,00	0,00
	Q9V1Z5	ERCC4/XPF	0,03	0,04
	Q9UYE0	Metallo-beta-lactamase pab1035	0,03	0,03
	Q9UZ60	minD/MRP superfamily ATPase	0,04	0,15
	Q9V076	Metallo-beta-lactamase pab1751	0,05	0,08
	Q9V2F8	Q9V2F8	0,06	0,11
	Q9V113	RNAP rpoA2	0,07	0,12
	Q9V2G3	RFC-ls	0,07	0,12
	Q9UYC4	ERCC3/XPB	0,08	0,15
	Q9V1X9	HsdS	0,09	0,13
	Q9UXY5	Histone acetyltransferase Elp3 homolog	0,09	0,11
	Q9V1F5	Archaeal histone B	0,09	0,08
	Q9V0B3	Adenine-specific DNA methylase	0,10	0,19
	Q9V2G4	RFC-ss	0,11	0,11
	Q9UZY0	N-glycosylase/DNA lyase/OGG1	0,12	0,19
	Q9UYC3	BAX1/Uncharacterized ArCR	0,13	0,13
	Q9UYB0	ApeH acylamino-acid-releasing enzyme	0,13	0,12
	Q9V135	Type II DNA topoisomerase VI subunit B	0,14	0,17
	Q9UZT4	Q9UZT4	0,15	0,18
	P61881	Archaeal histone A	0,15	0,15
	Q9V2K6	Q9V2K6	0,18	0,12
**Mre11/Rad50 complex**	Q9UZC8	Rad50	0,01	0,01
	Q9UYX8	PCNA	0,02	0,10
	Q9UZC9	Mre11	0,04	0,06
**RadA**	Q9V233	radA	0,01	0,01
	Q9V076	Metallo-beta-lactamase pab1751	0,09	0,14
	P0CL75	DNA ligase	0,12	0,09
**RadB**	Q9V2F6	radB	0,01	0,01
	Q9UYR4	Q9UYR4	0,13	0,20
	Q9UZT4	Q9UZT4	0,14	0,12
	P0CL75	DNA ligase	0,20	0,17
**Alka DNA glycosylase**	Q9UZ73	AlkA 3-methyladenine DNA glycosylase	0,00	0,00
	Q9V113	RNAP rpoA2	0,01	0,01
	Q9UZM4	Lhr-2 large helicase-related protein	0,02	0,01
	Q9V1X9	HsdS	0,02	0,01
	Q9V1Y1	HsdR	0,02	0,04
	Q9V2I6	Fructose-bisphosphate aldolase class 1	0,02	0,03
	Q9V1Z1	RPA32	0,02	0,07
	Q9V051	Putative ATPase of the AAA superfamily	0,03	0,03
	Q9V0V0	Glycerol-1-phosphate dehydrogenase	0,05	0,05
	Q9V2F9	RNA-binding protein AU-1	0,05	0,04
	Q9V2B6	Q9V2B6	0,05	0,05
	Q9UZ86	Reverse gyrase	0,05	0,05
	Q9V114	RNAP rpoA1	0,05	0,05
	Q9V076	Metallo-beta-lactamase pab1751	0,06	0,07
	Q9V2K6	Q9V2K6	0,06	0,07
	Q9UZB6	DNA helicase, putative	0,06	0,06
	Q9UYS8	DNA topoisomerase 1	0,07	0,05
	Q9UY25	Endonuclease IV	0,08	0,11
	Q9V1Y9	RPA41	0,09	0,13
	Q9V115	RNAP rpoB	0,09	0,08
	Q9UY23	RNAP rpoE1	0,10	0,13

	Q9UZP0	RNAP rpoF	0,10	0,11
	Q9UZ60	minD/MRP superfamily ATPase	0,10	0,14
	Q9UXY5	Histone acetyltransferase Elp3 homolog	0,10	0,09
	Q9V2J5	MoaB-1	0,10	0,07
	Q9V0W6	Endonuclease III	0,11	0,12
	Q9V1X8	HsdM	0,12	0,10
	Q9V291	DNA primase P46	0,13	0,12
	Q9UY16	Q9UY16	0,13	0,14
	Q9V2D7	Q9V2D7	0,14	0,10
	Q9UZD5	L-tyrosine decarboxylase	0,17	0,18
**OGG1 N-glycosylase/DNA lyase**	Q9V1Z0	RPA14	0,00	0,00
	Q9UY07	Nucleotidyltransferase	0,00	0,00
	Q9UZY0	N-glycosylase/DNA lyase/OGG1	0,01	0,01
	Q9UZM4	Lhr-2 large helicase-related protein	0,01	0,01
	Q9V1Z1	RPA32	0,01	0,01
	Q9UYS8	DNA topoisomerase 1	0,01	0,01
	Q9V0H2	Lhr-1 large helicase-related protein	0,02	0,02
	Q9V1Y9	RPA41	0,02	0,02
	Q9UZ86	Reverse gyrase	0,02	0,02
	Q9V113	RNAP rpoA2	0,03	0,03
	Q9V0W6	Endonuclease III	0,07	0,10
	Q9V2J5	MoaB-1	0,07	0,08
	Q9V292	DNA primase P41	0,07	0,08
	Q9V227	GptA purine phosphoribosyltransferase	0,08	0,07
	Q9UZP0	RNAP rpoF	0,08	0,10
	Q9UY23	RNAP rpoE1	0,09	0,10
	Q9V1N7	Methylated-DNA--protein-cysteine methyltransferase	0,09	0,11
	Q9UZX1	Q9UZX1	0,10	0,10
	Q9V0F2	putative Phosphoesterase	0,10	0,10
	Q9V2M4	RNAP rpoL	0,11	0,12
	Q9V0B1	Q9V0B1	0,12	0,10
	Q9V114	RNAP rpoA1	0,13	0,10
	Q9V116	RNAP rpoH	0,14	0,13
	Q9UZW9	Q9UZW9	0,16	0,20
	Q9V2B6	Q9V2B6	0,17	0,16
**NAD-dependant protein deacetylase**	Q9UZE7	NAD-dependant protein deacetylase	0,02	0,02
	Q9UYR4	Q9UYR4	0,10	0,12
**PHP domain containing protein**	Q9V0F9	Putative phosphotransferase, php family	0,00	0,00
	Q9UZT4	Q9UZT4	0,20	0,20
**Rad-25 like**	Q9V278	DNA repair protein RAD25	0,00	0,00
	Q9V113	RNAP rpoA2	0,02	0,01
	Q9V1Z1	RPA32	0,05	0,13
	Q9V2F9	RNA-binding protein AU-1	0,06	0,08
	Q9V114	RNAP rpoA1	0,07	0,07
	Q9UZP0	RNAP rpoF	0,10	0,16
	Q9V2B6	Q9V2B6	0,10	0,09
	Q9UY96	Putative flavoprotein monooxygenase	0,12	0,14
	Q9UZ86	Reverse gyrase	0,16	0,12
	Q9V115	RNAP rpoB	0,16	0,17
	Q9UY23	RNAP rpoE1	0,16	0,20
	Q9UZM4	Lhr-2 large helicase-related protein	0,17	0,04
